# Correction to “The Principle of Limb Reconstruction—“One Walking, Two Lines, and Three Balances”: A Retrospective Analysis of Post‐Traumatic Lower Limb Deformity Correction”

**DOI:** 10.1111/os.70241

**Published:** 2026-01-04

J. Zang, F. Wei, L. Shi, and S. Qin, “The Principle of Limb Reconstruction—“One Walking, Two Lines, and Three Balances”: A Retrospective Analysis of Post‐Traumatic Lower Limb Deformity Correction,” *Orthopaedic Surgery* 16 (2024): 2252–2263, https://doi.org/10.1111/os.14215.

The affiliation of Dr. Jiancheng Zang and Fang yuan Wei were incorrect, it should be:

1. Department of Foot and Hand Surgery, Beijing University of Chinese Medicine Third Affiliated Hospital, The Engineering Research Center “Traditional Chinese Medicine Orthopedics and Intelligent Rehabilitation”, Ministry of Education.

We apologize for this error.

